# Supplementary material for: Ecological Connectivity for Reptiles in Agroecosystems: A Case Study with Olive Groves in Liguria (Northwestern Italy)
Source: Animals (Basel). 2025 Mar 21;15(7):909. doi: 10.3390/ani15070909 (PMC11987836; doi:10.3390/ani15070909)
Supplement: Supplementary file 1 [file animals-15-00909-s001.zip › animals-3521538-supplementary.pdf]

Supplementary Materials to:

Ecological Connectivity for Reptiles in Agroecosystems: A Case Study with Olive Groves in Liguria (NW Italy)

Andrea Costa[1,*], Fabrizio Oneto[2], Giacomo Rosa[1], Giacomo Actis Dato[2] and Dario Ottonello [3]

[1] Department of Earth, Environmental and Life Sciences (DISTAV), University of Genova, Corso Europa 26, 16132 Genova, Italy
[2] Centro Studi Bionaturalistici (CeSBiN), Via San Vincenzo 2, 16121 Genova, Italy
[3] Agenzia Regionale per la Protezione dell'Ambiente Ligure (ARPAL), Via Bombrini 8, 16149 Genova, Italy
* Correspondence: andrea-costa-@hotmail.it

Table S1. List of natural protected areas included in the least cost path analysis for the two regions considered: Eastern Liguria and Western Liguria.

Id	Subregion	Protected Area code
W1	Western Liguria	IT07IT1316118
W2	Western Liguria	IT07IT1314609
W3	Western Liguria	IT07IT1314610
W4	Western Liguria	IT07IT1314611
W5	Western Liguria	IT07IT1314723
W6	Western Liguria	IT07IT1315313
W7	Western Liguria	IT07IT1315407
W8	Western Liguria	IT07IT1315408
W9	Western Liguria	IT07IT1315421
W10	Western Liguria	IT07IT1315503
W11	Western Liguria	IT07IT1315504
W12	Western Liguria	IT07IT1315602
W13	Western Liguria	IT07IT1315714
W14	Western Liguria	IT07IT1315719
W15	Western Liguria	IT07IT1315720
W16	Western Liguria	IT07IT1315805
W17	Western Liguria	IT07IT1315806
W18	Western Liguria	IT07IT1322326
W19	Western Liguria	IT07IT1323112
W20	Western Liguria	IT07IT1323201
W21	Western Liguria	IT07IT1323202
W22	Western Liguria	IT07IT1323203
W23	Western Liguria	IT07IT1323920
W24	Western Liguria	IT07IT1324011
W25	Western Liguria	IT07IT1324818
W26	Western Liguria	IT07IT1324896
W27	Western Liguria	IT07IT1324909
W28	Western Liguria	IT07IT1324910

W29	Western Liguria	IT07IT1314677
W30	Western Liguria	IT07IT1314678
W31	Western Liguria	IT07IT1314679
W32	Western Liguria	IT07IT1315380
E1	Eastern Liguria	IT07IT1344321
E2	Eastern Liguria	IT07IT1344323
E3	Eastern Liguria	IT07IT1344422
E4	Eastern Liguria	IT07IT1345005
E5	Eastern Liguria	IT07IT1345101
E6	Eastern Liguria	IT07IT1342806
E7	Eastern Liguria	IT07IT1343412
E8	Eastern Liguria	IT07IT1343415
E9	Eastern Liguria	IT07IT1343419
E10	Eastern Liguria	IT07IT1343502
E11	Eastern Liguria	IT07IT1343511
E12	Eastern Liguria	IT07IT1343520
E13	Eastern Liguria	IT07IT1343526
E14	Eastern Liguria	IT07IT1344210
E15	Eastern Liguria	IT07IT1344216
E16	Eastern Liguria	IT07IT1331718
E17	Eastern Liguria	IT07IT1331721
E18	Eastern Liguria	IT07IT1331810
E19	Eastern Liguria	IT07IT1331811
E20	Eastern Liguria	IT07IT1331909
E21	Eastern Liguria	IT07IT1332603
E22	Eastern Liguria	IT07IT1332614
E23	Eastern Liguria	IT07IT1332622
E24	Eastern Liguria	IT07IT1332717
E25	Eastern Liguria	IT07IT1333307
E26	Eastern Liguria	IT07IT1333308
E27	Eastern Liguria	IT07IT1333316
E28	Eastern Liguria	IT07IT1345109
E29	Eastern Liguria	IT07IT1345114

Table S2. Maxent model performance (AUC) and variable percent contribution for all modelled taxa

Taxon	AUC	Variable percent contribution in the Maxent model					
		Elevation	TPI	Insolation	Grasslands	TCD	SWF
Euleptes europaea	0.934	28.8	5.6	0.0	0.1	20.2	45.3
Hemidactylus turcicus	0.909	57.4	19.7	0.1	2.3	19.9	0.7
Tarentola mauritanica	0.894	54.5	16.4	2.1	2.1	20.1	4.8
Chalcides sp.	0.764	19.6	3.5	16.5	0.2	42.2	18
Lacerta bilineata	0.793	35.1	3.6	13.2	0.0	45.2	2.9
Podarcis sp.	0.782	34.0	4.3	9.8	2.6	46.0	3.2
Timon lepidus	0.858	4.2	35.8	12.3	2.0	37.1	8.6
Anguis veronensis	0.778	18.4	10.7	4.4	1.4	50.4	14.8
Coronella sp.	0.783	26.3	10	2.2	0.8	50.4	10.3
Hierophis viridiflavus	0.882	20.8	3.7	9.9	0.1	62.4	3.2
Malpolon monspessulanus	0.895	54.4	6.9	1.3	1.4	26.6	9.5
Natrix sp.	0.838	17.5	43.9	7.5	0.9	28.5	1.8
Zamenis longissimus	0.780	6.9	16.5	8.7	0.0	59.4	8.6
Vipera aspis	0.832	58	0.3	6.8	3.5	31	0.4
